# Roles of N^6^-methyladenosine (m^6^A) modifications in gynecologic cancers: mechanisms and therapeutic targeting

**DOI:** 10.1186/s40164-022-00357-z

**Published:** 2022-11-12

**Authors:** Jiahua Chen, Bao Guo, Xiaojing Liu, Jing Zhang, Junhui Zhang, Yuan Fang, Suding Zhu, Bing Wei, Yunxia Cao, Lei Zhan

**Affiliations:** 1 Obstetrics and Gynecology, The Second Affiliated Hospital of Anhui Medical University, No. 678 Furong Road, Hefei, 230601 Anhui China; 2 Department of Obstetrics and Gynecology, The First Affiliated Hospital of Anhui Medical University, No. 218 Jixi Road, Hefei, 230022 Anhui China

**Keywords:** N^6^-methyladenosine, Gynecologic cancer, Tumor microenvironment, Prognosis, Treatment

## Abstract

Uterine and ovarian cancers are the most common gynecologic cancers. N^6−^methyladenosine (m^6^A), an important internal RNA modification in higher eukaryotes, has recently become a hot topic in epigenetic studies. Numerous studies have revealed that the m^6^A-related regulatory factors regulate the occurrence and metastasis of tumors and drug resistance through various mechanisms. The m^6^A-related regulatory factors can also be used as therapeutic targets and biomarkers for the early diagnosis of cancers, including gynecologic cancers. This review discusses the role of m^6^A in gynecologic cancers and summarizes the recent advancements in m^6^A modification in gynecologic cancers to improve the understanding of the occurrence, diagnosis, treatment, and prognosis of gynecologic cancers.

## Background

The main types of gynecologic cancers, which seriously damage the female reproductive organs, include vulvar cancer, vaginal cancer, cervical cancer (CC), endometrial cancer (EC), uterine cancer, and ovarian cancer (OC). CC and OC are the most frequent types of gynecologic cancer, accounting for 6.5% and 3.4%, respectively, of all the new cancers in women [1]. A population-based study conducted on the epidemiological trends of gynecologic cancer from 1990 to 2019 indicated that the incidence and mortality of gynecologic cancers might have geographical variations and changes along with sociodemographic index value [2]. Most gynecologic cancer patients have no distinct symptoms or physical signs in the early stages. In addition, the specific biomarkers for the early diagnosis of gynecologic cancer are also lacking. Moreover, most of the cases are in advanced stages at the time of diagnosis. Therefore, understanding the pathogenesis of gynecologic cancer is particularly important. This might identify specific markers for early diagnosis and therapeutic targets for the related therapeutic drugs, thereby ultimately improving the prognosis and quality of patients [3–5].

N^6^-methyladenosine (m^6^A) was first discovered in 1974 as an internal chemical modification, which was widely observed in the messenger RNAs (mRNAs) and non-coding RNAs (ncRNAs) [6]. The m^6^A plays important role in numerous aspects of RNA metabolism, such as pre-mRNA splicing, processing of 3′-untranslated region (UTR), export, translation, and degradation of mRNA, and processing of non-coding RNA [7–10]. Recent studies have shown that the m^6^A regulatory proteins act as writers, erasers, and readers, thereby modulating the dynamic deposition of mRNAs and other nuclear RNAs [11, 12]. These findings strongly suggest the dynamic regulatory role of m^6^A modification is similar to the other well-known chromosomal reversible epigenetic modifications. This reversible RNA methylation provides a new dimension in the post-transcriptional regulation of gene expression [11].

In addition to mRNAs, m^6^A is also reported in a variety of ncRNAs, such as microRNAs (miRNAs), long non-coding RNAs (lncRNAs), circular RNAs (circRNAs), and ribosomal RNAs (rRNAs) and has been indicated to be crucial for their metabolism and function [13–15]. In addition, abnormal m^6^A modifications in ncRNA by some m^6^A regulatory proteins participate in the proliferation, invasion, and drug resistance of cancer cells, thereby indicating their potential association with cancer [16, 17]. Therefore, this new field in cancer pathogenesis might provide new opportunities for the diagnosis and treatment of cancer.

This review summarizes the recent studies on m^6^A modifications in OC, CC, and EC, particularly focusing on the regulatory mechanism of m^6^A regulatory proteins in promoting the proliferation, invasion, and metastasis of these three gynecologic cancers. Finally, the current knowledge and prospects of m^6^A modifications in the tumor immune microenvironment, diagnosis, and prognosis of gynecologic cancer are also discussed.

## m^6^A modification

The m^6^A methylase complex consists of at least five methyltransferases (writers) with methyltransferase-like 3 (METTL3) as its catalytic core. METTL14 is present as the structural support for METTL3; this core complex is stabilized by wilm’s tumor-associated protein (WTAP). The RNA binding pattern protein 15 (RBM15) helps the recruitment of the complex to its target site. Another component of this complex is vir-like m^6^A methyltransferase-associated protein (VIRMA), which is also known as KIAA1429; its molecular function is uncertain [18]. On the other hand, a demethylase (eraser), consisting of fat mass and obesity-associated protein (FTO) and alkB homolog 5 (ALKBH5), removes the m^6^A modifications, thereby reducing its modification rate [19, 20]. The functional interaction between the methyltransferases and demethylase of m^6^A might contribute to the dynamic regulation of the m^6^A modifications.

Recent studies have identified the m^6^A binding proteins (readers) for mRNA, containing YT521-B homology (YTH) domains, such as YTHDF1-3, YTHDC1, and YTHDC2, which have shown a greater affinity for the methylated mRNAs as compared to the non-methylated mRNA [21–25]. If induced by the different cellular environments, the YTH proteins, belonging to the same YTH domain family, can bind to the different subsets of the m^6^A site and regulate different genes [21]. For instance, the YTHDF2 protein colocalizes with the decay factor and enhances the m^6^A-modified mRNAs expression [26, 27]. On the other hand, YTHDF1 can bind to the m^6^A site near the stop codon, thereby subsequently activating the translation by interacting with eukaryotic translation initiation factor 3 (eIF3) [28]. Another YTH protein, YTHDF3 can decay mRNA when working with the YTHDF2 while enhancing the translation of m^6^A-modified RNA when working with YTHDF1 [10, 29].

## m^6^A and OC

**Fig. 1:**
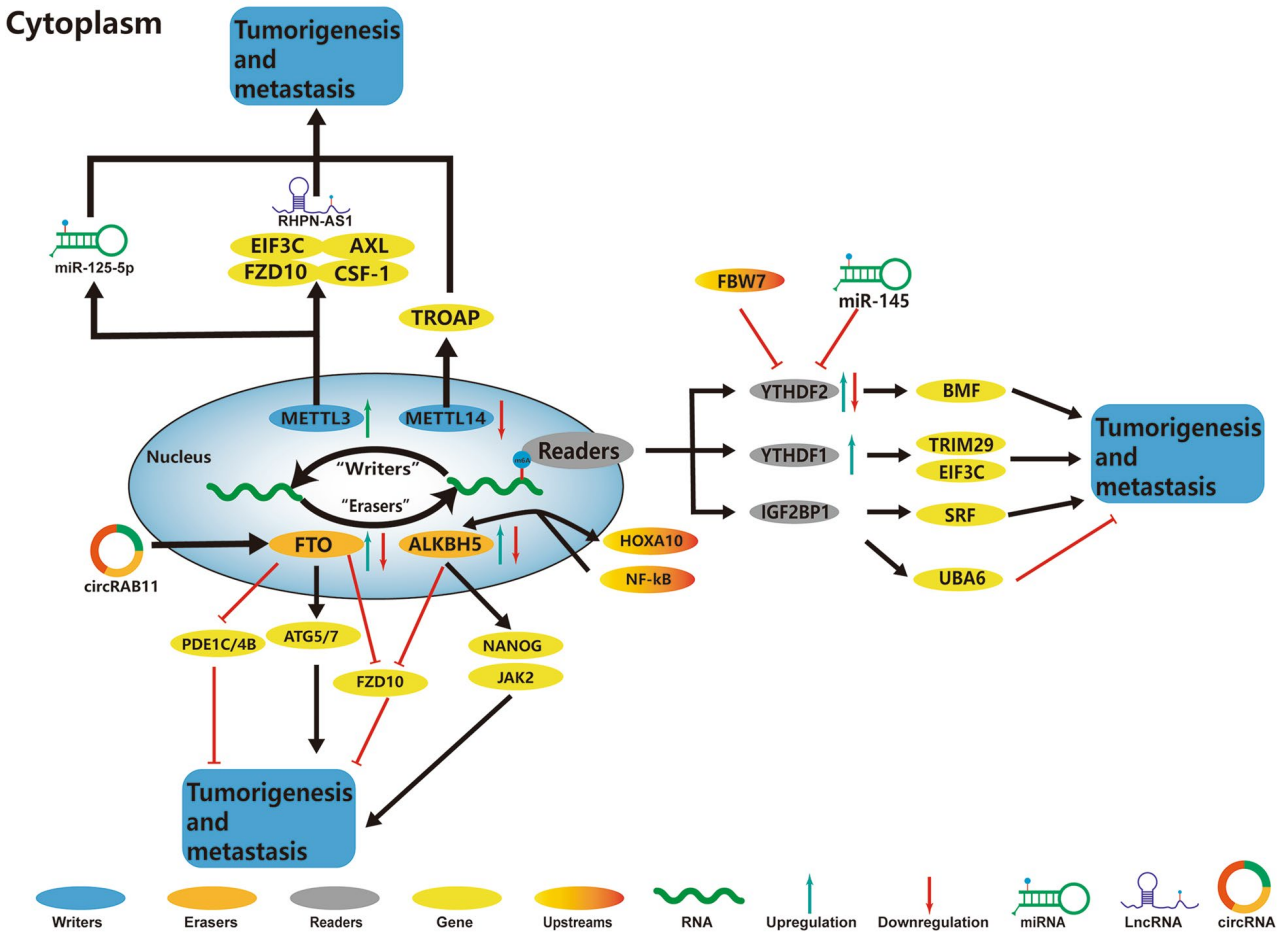
In OC, m^6^A regulatory proteins contribute to tumorigenesis and metastasis by interacting with various RNAs. METTL3 and METTL14 stimulate the progression of OC by promoting the expression levels of FZD10, CSF-1, EIF3C, AXL, RHPN-AS1, miR-125-5p, and TROAP. HOXA10 forms a loop with ALKBH5 and jointly activates the JAK2/STAT3 signaling pathway by mediating JAK2 m^6^A methylation and promoting the OC resistance to cisplatin. The activated NF-κB up-regulates ALKBH5 expression and increases m^6^A level and NANOG expression, contributing to ovarian carcinogenesis**.** FTO and ALKBH5 stimulate/inhibit the progression of OC by affecting the expression levels of ATG5, ATG7, PDE1C, PDE4B, and FZD10.YTHDF1, YTHDF2 and IGF2BP1 stimulate/inhibit the progression of OC by affecting the expression of BMF, TRIM29, EIF3C, SRF, and UBA6. In addition, FBW7 and miR-145 inhibit the expression of YTHDF, leading to OC suppression

Numerous studies elucidated that the m^6^A regulators could participate in many functions in OC, such as dysregulation of signaling pathways and anti-tumor drug resistance. The role and mechanism of m^6^A regulators in OC are summarized in Fig. 1 and Table 1.

**Table 1:** The role and mechanism of m^6^A regulators in OC

m^6^A regulators	Roles	Genes/RNAs	Mechanisms	Model	Refs.
*Writers*					
METTL3	Oncogene	AXL	METTL3 stimulates AXL translation and EMT	In vitro; in vivo	[30]
METTL3	Oncogene	AKT	METTL3 regulates the phosphorylation levels of AKT and the expression of the downstream effector Cyclin D1	In vitro	[32]
METTL3	Oncogene	miR-126-5p	METTL3 promotes miR-126-5p maturation, leading to the activation of PTEN-mediated PI3K/Akt/mTOR pathway	In vitro; in vivo	[33]
METTL3	Oncogene	RHPN1-AS1	METTL3 increases m^6^A level of lncRNA RHPN1-AS1 and contributes to its stability	In vitro; in vivo	[37]
METTL14	Tumor suppressor	TROAP	METTL14 negatively regulates TROAP expression in an m^6^A-dependent manner	In vitro; in vivo	[40]
*Readers*					
YTHDF1	Oncogene	TRIM29	YTHDF1 promotes TRIM29 translation, then affecting the CSC-like characteristics of OC cells	In vitro	[49]
YTHDF1	Oncogene	EIF3C	YTHDF1 promotes EIF3C’s translation in an m^6^A-dependent manner and affects the overall protein translation	In vitro; in vivo	[47]
YTHDF2	Oncogene	miR-145	A double negative feedback loop between miR-145 and YTHDF2, regulates the proliferation and migration of OC cells	In vitro	[48]
YTHDF2	Oncogene	FBW7	YTHDF2 is suppressed by FBW7, recognizes m^6^A-modifed BMF mRNA and accelerates decay of the latter	In vitro; in vivo	[45]
IGF2BP1	Oncogene	SRF	IGF2BP1 promotes SRF expression to augment SRF-dependent transcription	In vitro	[46]
IGF2BP1	Tumor suppressor	UBA6	IGF2BP1 enhances the stability of UBA6 mRNA, thus inhibiting the malignancy of OC	In vitro	[51]
*Erasers*					
FTO	Tumor suppressor	PDE1C PDE4B	FTO reduces m^6^A level at the 3’UTR and the mRNA stability of PDE1C and PDE4B, thus blocking cAMP pathway	In vitro; In vivo	[53]
FTO	unknown	ATG5/7	circRAB11FIP1 promotes autophagy through FTO-mediated demethylation of ATG5 and ATG7	In vitro	[57]
*Erasers*					
FTO/ALKBH5	unknown	FZD10	Downregulation of ALKBH5 and FTO contribute to PARPi resistance by increasing m^6^A modification in FZD10 mRNA to upregulate Wnt signaling	In vitro; in vivo	[56]
ALKBH5	Oncogene	NANOG	Highly expressed TLR4 activates NF-κB pathway, upregulates ALKBH5 expression and increases m^6^A level and NANOG expression	In vitro; in vivo	[54]
ALKBH5	Oncogene	JAK2	ALKBH5-HOXA10 loop jointly activates the JAK2/STAT3 signaling pathway by mediating JAK2 m^6^A demethylation	In vitro; in vivo	[55]
*Immunoregulators*					
ZC3H13/YTHDF1/IGF2BP1	NA	NA	Important immune cell infiltration-regulated m^6^A regulators	NA	[79]

*NA* not available

### Role of m^6^A regulators in the progression of OC

#### m^6^A writer and OC

Studies on OC have mainly focused on the regulatory factor METTL3 [30–33]. Numerous studies have shown that METTL3 plays an important role in the tumorigeneses of lung and hepatocellular cancer (HCC) [14, 34, 35]. However, METTL3 has also been identified as a tumor suppressor in renal cell carcinoma (RCC) and inhibits the proliferation, migration, and progression of cancer [36]. Ma et al. first reported that METTL3 could promote m^6^A methylation in the OC without interacting with METTL14 and WTAP [31]. A previous study also showed that in human cancer, METTL3 could directly regulate the specific mRNA translation by recruiting eIF3 without coordinating with METTL14 and WTAP [34]. Studies suggested that METTL3 had a novel m^6^A regulatory mechanism, which might play an important function in the occurrence and development of OC. Currently, numerous studies indicate that METTL3 can promote the occurrence of OC by affecting the maturation and stability of multiple RNAs. Hua et al. first confirmed the role of METTL3 in tumor progression in the OC cells [30]. In-vivo and in-vitro studies reported that METTL3 could promote the epithelial-to-mesenchymal transition (EMT) by stimulating the mRNA translation of AXL receptor tyrosine kinase (AXL), which might play an important role in the occurrence and/or invasion of OC [30]. Further studies showed that there were no significant changes in the expression levels of AXL in YTHDF1-silenced cells [30]. A study suggested that METTL3 might act as an m^6^A reader, thereby directly promoting the translation of mRNA; these results were consistent with a previous study, which reported that METTL3 could promote the translation of mRNA in the human lung cancer cells, containing m^6^A regulatory proteins in their cytoplasm [30, 34]. However, the exact mechanism of the role of m^6^A requires further investigation.

Liang et al. reported that knocking down the METTL3 gene decreased the expression levels of phosphorylated AKT and its downstream p70S6K and cyclin D1, indicating a decrease in the activation of the AKT pathway in the absence of METTL3 [32]. Bi et al. confirmed that METTL3 could regulate the m^6^A level of miR-126-5p, promoting its maturation which activated the phosphoinositide 3-kinase (PI3K)/Protein kinase B (AKT)/mechanistic target of rapamycin (mTOR) signaling pathway [33]. In addition, in a xenograft experiment, the knockdown of phosphatase and tensin homolog (PTEN) gene could reverse the METTL3 knockdown-induced decrease in the expression levels of PI3K, p-AKT, and p-4EBP1. The results further confirmed that the knockdown of the METTL3 gene inhibited the PI3K/AKT/mTOR signaling pathway by inhibiting the expression of miR-126-5p and upregulating that of PTEN in-vivo. These results showed that silencing the METTL3 gene could decrease tumor growth and PTEN gene silencing [33]. METTL3 could improve the m^6^A modification levels in RHPN1-antisense RNA 1(RHPN1-AS1) and increase its RNA stability, which might partially contribute to the up-regulation of RHPN1-AS1 in OC [37]. RHPN1-AS1 absorbs miR-596, which increases the expression of LETM1 and activates the FAK/PI3K/AKT signaling pathway, thereby contributing to the occurrence and development of cancer [37]. These results were similar to Luo’s study, which indicated that YTHDF1 could promote the HCC progression by activating the PI3K/AKT/mTOR signaling pathway and inducing EMT [38].

In most tumors, METTL14 downregulates the m^6^A levels in cancer cells by acting as m^6^A methyltransferase to inhibit the occurrence and development of tumors, thereby playing an anti-tumor role. In breast cancer (BC), the low METTL14 level was correlated with a poor prognosis, and its abnormal expression could promote the invasion of BC by affecting the tumor progression-related pathways and mediating the immunosuppression [39]. The studies on OC have shown similar results. Liang et al. reported a considerable copy number variation (CNV) in the METTL14 gene in the OC tissues and a decrease in its expression level as well as low m^6^A methylation level [40]. Further investigation showed that the trophinin-associated protein (TROAP) was a downstream target of METTL14 [40]. METTL14 reduced the mRNA stability of TROAP, inhibiting the proliferation of OC cells at the G1 phase [40].

In addition, WTAP, an m^6^A writer, has been reported as a classic biomarker for the progression and metastasis of OC, thereby contributing to the diagnosis and prognosis of OC [41, 42]. Fu et al. reported that the overexpression of WTAP in the OC cells resulted in a high malignancy and low survival rate and promoted aberrant methylation of mRNA, thereby regulating the growth and migration of tumor cells [43]. Wang et al. reported a positive correlation of WTAP expression with two genes, including a family with sequence similarity 76 member A (FAM76A) and HBS1-like translational GTPase (HBS1L) [44]. However, the mechanism of the WTAP-HBS1L/ FAM76A axis, playing a functional role in the progression of OC, remains unclear.

#### m^6^A reader and OC

In OC, numerous studies have elucidated the different functional roles of YTHDF1, YTHDF2, and Insulin-like growth factor 2 mRNA-binding proteins (IGF2BPs) [45–49]. YTHDF1, a YTH domain family member, can identify the m^6^A post-transcriptional modification by the conserved aromatic cages in its YTH domain [50]. All the YTH domain-containing proteins can bind to the m^6^A sites in mRNA; however, they recognize different target mRNAs and play different functional roles. Studies have shown an increase in the recruitment of tripartite motif-containing 29 (TRIM29) mRNA by YTHDF1 in the cisplatin-resistant OC cells, thereby promoting the translation of TRIM29 transcripts [49]. Knocking down the YTHDF1gene inhibited the cancer stem cell (CSC)-like characteristics in the cisplatin-resistant OC cells, which were rescued by the overexpression of TRIM29 gene, thereby suggesting its oncogenic role in an m^6^A-YTHDF1-dependent manner [49]. Based on the multi-group analysis of OC, researchers have identified a new mechanism, involving EIF3C, a subunit of the translation initiation factor [47]. YTHDF1 could bind to the m^6^A-modified mRNA of EIF3C, stimulate the EIF3C translation, and promote its overall translational output, ultimately leading to the progression and metastasis of OC [47]. Recent studies have reported that the IGF2BPs in m^6^A “reader” also plays a similar role. Müller et al. found that IGF2BP1 could downregulate the mRNA expression of serum response factor (SRF) mediated by the miRNA production, thereby promoting the expression of SRF in an m^6^A-dependent manner [46]. Wang et al. also identified IGF2BP1 as an m^6^A reader of ubiquitin-like modifier activating enzyme 6 antisense RNA 1 (UBA6-AS1) -RBM15, which mediated the m^6^A mRNA level of UBA6, thereby enhancing its stability; this ultimately inhibited the proliferation, migration, and invasion of OC cells via UBA6 [51]. These studies suggested that YTHDF1 and IGF2BPs were involved in the OC progression by increasing the translation of target mRNA.

Recent studies demonstrated a novel tumor-promoting effect of YTHDF2 by analyzing its upstream signal in the OC and showed that the chemical modification of m^6^A as a signal center could interact with important metabolic pathways [45, 48]. Li et al. reported a close correlation between an increase in the YTHDF2 protein levels and the OC tissues in a clinical setting [48]. In addition, the investigation showed a key crosstalk between the miR-145 and YTHDF2 via a double-negative feedback loop [48]. Researchers have also reported similar double-negative feedback circuits in the HCC. The miR-145, which was down-regulated in the HCC patients, could directly target the 3′-UTR of YTHDF2 mRNA, thereby inhibiting its expression [52]. Additionally, Xu et al. demonstrated the E3-ubiquitin ligase F-box and WD repeat domain-containing 7 (FBW7), a tumor suppressor, could degrade YTHDF2 in the OC [45]. This study depicted the mechanism of YTHDF2 and FBW7 in OC; FBW7 could decrease the YTHDF2-mediated m^6^A-dependent mRNA decay for stabilizing the pro-apoptotic BMF mRNA [45]. In particular, Xu et al. demonstrated the role of the FBW7-YTHDF2-BMF axis in the occurrence and development of OC and described how the m^6^A-related regulatory factors in OC were activated, which provided new insights into the mechanism of OC.

#### m^6^A eraser and OC

In OC, studies on m^6^A erasers have focused on FTO and ALKBH5 [53–57]. The FTO gene is associated with obesity in children and adults [58] and regulates energy homeostasis by controlling food intake and fine-tuning nutritional sensing at the cellular level [59]. FTO is the first recognized nucleic acid demethylase, which physiologically targets the m^6^A residues in mRNA [19, 60]. Studies have reported its high expression in acute myeloid leukemia (AML), promoting cellular transformation and proliferation by the post-transcriptional regulation of ASB2, RARA, MYC, and CEBPA [61, 62]. CSCs have the abilities of self-renewal, spherical growth, differentiation and tumor formation, which are correlated with the initiation, metastasis, and recurrence of high-grade serous OC (HGSOC) after chemotherapy [63, 64]. Interestingly, Huang et al. recently reported a tumor-inhibitory effect of FTO in HGSOC, which was contradictory to the tumor-promoting role of the FTO previously reported in other types of cancer. Huang et al. demonstrated that FTO targeted two phosphodiesterase genes (PDE4B and PDE1C), thereby regulating the cAMP signal transduction and maintaining the stemness of ovarian CSCs [53]. Their study demonstrated the inhibitory effects of FTO on solid tumors for the first time and identified the cAMP signal transduction as a key pathway for the self-renewal of CSCs regulated by m^6^A mRNA modification [53]. In addition, another eraser ALKHB5 was also demonstrated to upregulate the expression level of NANOG [54]. A study showed that ALKBH5 could mediate the post-transcriptional NANOG expression and enrich CSCs in BC [65]. ALKBH5 showed significantly higher expression levels in OC tissues as compared to the normal ovarian tissues; however, its expression in the OC cell lines was lower than that in the normal ovarian cells in-vitro [54]. Jiang et al. reported similar expression patterns for the Toll-like receptor 4 (TLR4) in the tumor microenvironment (TME) [54]. Further investigation verified that the highly expressed TLR4 could activate the nuclear factor kappa B (NF-κB) pathway, upregulate the ALKBH5 expression, and increase the m^6^A level and NANOG expression, thereby contributing to the tumorigenesis of OC [54]. Their study revealed an important functional role of mRNA m^6^A modification in the self-renewal of OC cells, particularly in the TME.

Autophagy is related to the status of physiological processes and is involved in many pathological conditions, including tumors and inflammation. Currently, numerous studies have confirmed the effects of circRNA on the malignant behavior of tumor cells by regulating autophagy. For example, CircDnmt1 stimulated autophagy in the BC cells to promote their proliferation [66]. CircRNA 103948 acted as competing endogenous RNA (ceRNA) and inhibited autophagy in CRC [67]. CircRNA ST3GAL6 could inhibit the malignant behavior of gastric cancer by regulating autophagy through the FOXP2/MET/mTOR axis [68]. Interestingly, Zhang et al. reported that the m^6^A modification was involved in autophagy [57]. They demonstrated that CircRAB11FIP1 could bind to the mRNA of FTO, promoting its expression, and thereby regulating the m^6^A methylation level of ATG5 and ATG7 mRNA through FTO, which ultimately promoted the autophagy and malignant behavior of OC [57].

Tumors show resistance to anti-tumor drugs by numerous mechanisms, including mutation of tumor suppressor genes, activation of oncogenes, and dysregulation of the signaling pathways [69–71]. In OC, the FTO and ALKBH5 are involved in drug resistance by activating or upregulating a specific signaling pathway [55, 56]. Takeshi et al. demonstrated a significant increase in m^6^A modification in mRNA frizzled class receptor 10 (FZD10), thereby increasing its stability and upregulating the Wnt/β-catenin pathway [56]. Further investigation showed that the downregulation of m^6^A demethylases FTO and ALKBH5 enhanced the m^6^A modification of FZD10 mRNA and reduced the sensitivity of PARP inhibitor (PARPi), which increased the activity of homologous recombination [56]. Another study reported that ALKBH5- Homeobox A10 (HOXA10) loop could mediate the demethylation of Janus kinase 2 (JAK2) m^6^A and cisplatin resistance in the OC [55]. Noteworthily, HOXA10 could form a loop with ALKBH5 and act as an upstream transcription factor of ALKBH5. Its overexpression could also enhance chemoresistance in the OC cells [55]. The activation of the JAK2/STAT3 signaling pathway can result in the overexpression of the ALKBH5-HOXA10 loop [55]. These studies have explained the mechanism of m^6^A modification in the anti-tumor drug resistance of OC. However, more detailed studies are needed in the future to study the mechanism in detail.

### m^6^A and immunoregulation in OC

TME includes tumors, surrounding matrix, and immune components, such as tumor-associated macrophages (TAMs), CD8^+^ T lymphocytes, and myeloid-derived suppressor cells (MDSCs) [72]. Recent studies have demonstrated numerous important roles of TME components in various biological behaviors of cancer, such as invasion, metastasis, and immune evasion from immune surveillance [73, 74]. Currently, based on the level of tumor-infiltrating immune cells (TICs) in TME, tumors have been classified into two groups: hot tumors, containing high-density CD8+ T lymphocytes, and cold tumors, lacking T lymphocytes [75–77]. Despite the relatively high tumor mutation burden in OC, it is a cold tumor, generally lacking the infiltration of cytotoxic T lymphocytes, and thereby lacking the ability to recognize all the tumor antigens [78]. Numerous studies have demonstrated that the degree of immune cell infiltration and expression levels of various immune gene markers are correlated with the specific m^6^A regulators [54, 79–81]. Wang et al. reported RBM15B, Zinc finger CCCH domain-containing protein 13 (ZC3H13), YTHDF1, and IGF2BP1 as important immune cell infiltration-regulated m^6^A regulators in the OC [79]. Yan et al. also reported cell division cycle 42 effector protein 3 (CDC42EP3) as a possible target gene of m^6^A, which was downregulated by m^6^A regulators in the OC cells and tissues [80]. Interestingly, the expression of CDC42EP3 was reported to be correlated with the various TICs, including natural killer cells, T central memory cells, and T gamma delta cells [80]. Jiang et al. further elucidated the mechanism of m^6^A regulatory factors involved in immune responses [54]. The m^6^A eraser ALKBH5 could promote the development of OC by stimulating the NF-κB pathway in the TME [54].

### Prognostic effect of m6A in OC

Numerous OC patients are diagnosed in the advanced stages due to the lack of specific biomarkers for early clinical screening as well as due to their relatively nonspecific disease symptoms. Currently, more than 70% of OC patients show < 30% overall survival after five years of cytoreductive surgery and adjuvant chemotherapies [82]. In order to better control tumorigenesis and monitor the prognosis of OC patients, new factors and biomarkers are needed to be developed.

Advancements in the studies of m^6^A methylation in OC might elaborate the prognostic potential of m^6^A regulatory factors and m^6^A-related genes in OC. Recently, numerous studies identified that the m^6^A RNA methylation regulator exhibited high frequencies of genetic changes and high prognostic potential in OC (Table 4) [83–87]. Han et al. reported the correlation of genetic mutations in patients with OC with the survival and m^6^A regulator genes [84]. Zhu et al. reported the association of YTHDC2 and KIAA1429 with the prognosis of OC patients [88]. In addition, researchers have established genetic models to predict the prognosis of OC patients. Fan et al. established a genetic model, consisting of three m^6^A regulatory genes, which could be used to predict the progression of OC patients [86]. Li et al. also established a risk-scoring model, consisting of three m^6^A RNA methylation regulators (VIRMA, IGF2BP1, and HNRNPA2B1) and a related miRNA-m^6^A regulator-m^6^A target gene network [87]. Similarly, Jiao et al. also established a genetic model, containing 12 genes (WTAP, LGR6, ZC2HC1A, SLC4A8, AP2A1, NRAS, CUX1, HDAC1, CD79A, ACE2, FLG2, and LRFN1) [89]. Moreover, several studies also have verified the reliability of these results. For example, Yu et al. reported the correlation of high WTAP expression with poor prognosis in HGSOC [90]. However, different m^6^A regulatory genes have shown different expression patterns in the different tumor types or independent databases, further demonstrating the complexity of the m^6^A post-transcriptional regulation mechanism and suggesting the tumor-specificity of m^6^A regulatory factors.

LncRNAs are a group of 200-nucleotides long RNA, which regulate gene expression and various physiological and pathological processes [91, 92]. Using the OC-related dataset from The Cancer Genome Atlas (TCGA), Nie et al. confirmed the prognostic potential of 21 m^6^A modifications in OC and identified two m^6^A subtypes using the m^6^A-related gene expression profiles [93]. Then, the authors established an OC risk model based on the differential expression pattern of lncRNAs between the m^6^A subtypes and lncRNAs co-expressed with the m^6^A-related genes [93]. The risk model not only simply evaluated the predictability of tumor prognosis using the risk score but also assessed the effectiveness of immunotherapy and developed novel and more accurate immunotherapies.

## m^6^A and CC

**Fig. 2:**
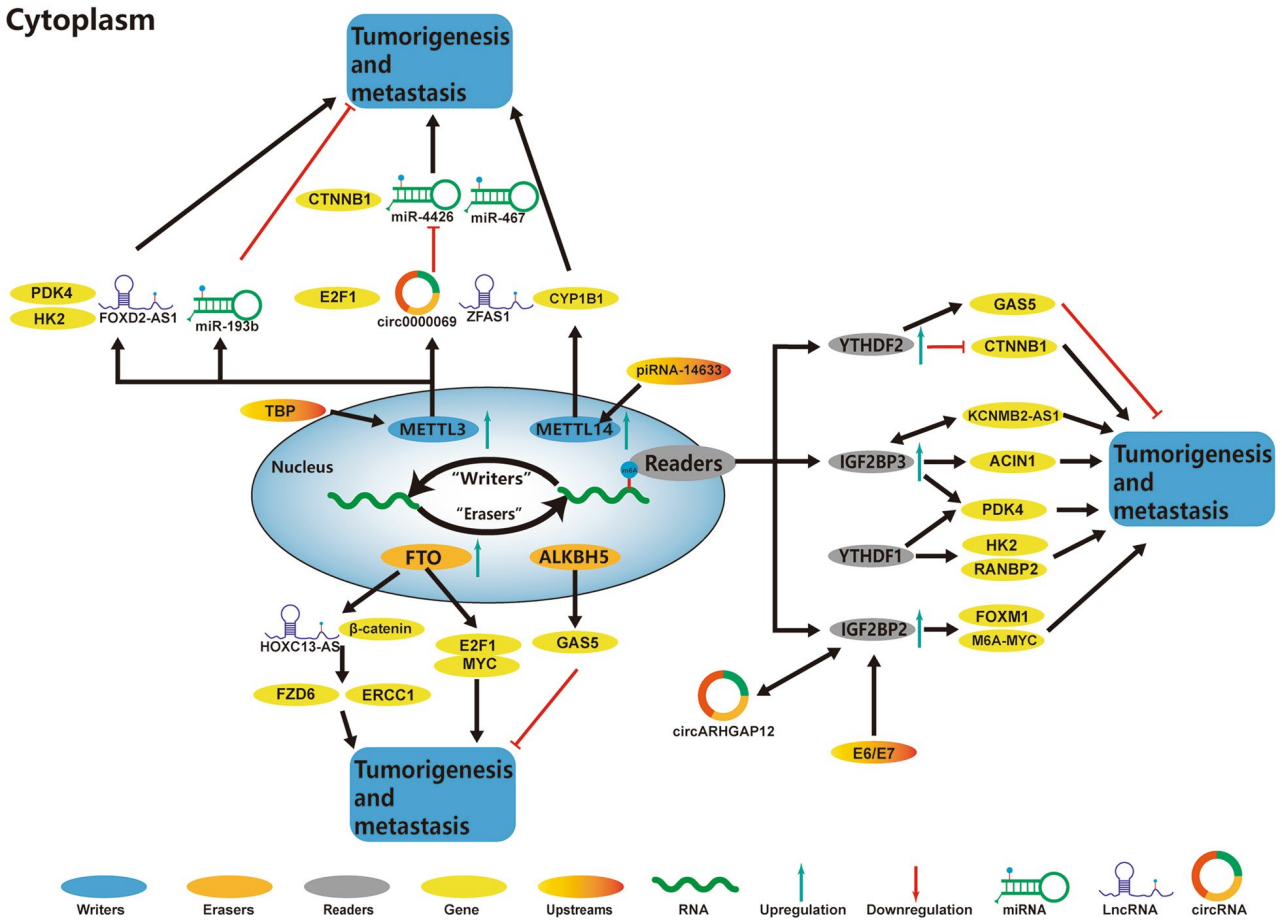
In CC, m^6^A regulatory proteins contribute to tumorigenesis and metastasis by interacting with various RNAs. piRNA-14633 mimic increases the mRNA stability of METTL14 and CYP1B1 expression levels, leading to the malignancy of CC. METTL3, which is up-regulated by TBP, regulates the glycolysis of CC via the regulation of PDK4. METTL3 promotes/inhibits the tumorigenesis and metastasis of CC by interacting with various RNAs, including E2F1, circ0000069, ZFAS1, miR-193b, FOXD2-AS1, and HK2. FTO and ALKBH5 promote/inhibit the tumorigenesis and metastasis of CC by regulating the expression of β-catenin, HOXC13-AS, E2F1, MYC, and GAS5. circARHGAP12 interacts with IGF2BP2 to enhance the mRNA stability of FOXM1, thereby promoting the proliferation and migration of CC. E6/E7 regulates the aerobic glycolysis of CC cells through the IGF2BP2-mediated modulation of m^6^A-MYC mRNA. KCNMB2-AS1 and IGF2BP3 form a positive regulatory circuit, which increases the tumorigenic effect of KCNMB2-AS1 in CC. YTHDF1, YTHDF2, and IGF2BP3 promote/inhibit the tumorigenesis and metastasis of CC by regulating the expression levels of GAS5, CTNNB1, ACIN1, PDK4, HK2, and RANBP2

Numerous studies have elucidated the participation of m^6^A regulators in many functions in the CC, such as aerobic glycolysis and EMT. The role and mechanism of m^6^A regulators in CC are summarized in Fig. 2 and Table 2.

**Table 2:** The role and mechanism of m^6^A regulators in CC

m^6^A regulators	Roles	Genes/RNAs	Mechanisms	Model	Refs.
*Writers*					
METTL3	Oncogene	miR-193b	METTL3 downregulates miR-193b to promote CC aggressiveness by targeting CCND1	In vitro; in vivo	[99]
METTL3	Oncogene	ACIN1	METTL3 interacts with IGF2BP3 to promote the mRNA stability of ACIN1	In vitro; in vivo	[100]
METTL3	Oncogene	β-catenin	METTL3 indirectly suppresses CTNNB1 transcription via stabilizing its transcription suppressor E2F1 mRNA; METTL3 represses membrane localization of β-catenin and its interaction with E-Cadherin by downregulating c-Met kinase	In vitro; in vivo	[101]
METTL3	Oncogene	circ0000069	circ0000069 is upregulated partially due to m^6^A modification, which promotes cell proliferation and migration via sponging miR-4426 in CC	In vitro; in vivo	[107]
METTL3	Oncogene	FOXD2-AS1	METTL3/FOXD2-AS1 promotes the tumorigenesis of cervical cancer through the LSD1/p21 axis	In vitro; in vivo	[110]
METTL3	Oncogene	ZFAS1	ZAFS1 sequesters miR-647, and this RNA–RNA interaction is regulated by METLL3-mediated m^6^A modification	In vitro; in vivo	[111]
METTL3	Oncogene	HK2	METTL3 recruits YTHDF1 to enhance HK2 stability, there by promoting the Warburg effect of CC	In vitro; in vivo	[118]
METTL14	Oncogene	CYP1B1	piRNA-14633 promotes proliferation, migration and invasion of CC cells by METTL14/CYP1B1 signaling axis	In vitro; in vivo	[106]
*Readers*					
YTHDF1	Oncogene	RANBP2	YTHDF1 regulates RANBP2 translation in an m^6^A-dependent manner without effect on its mRNA expression	In vitro; in vivo	[120]
IGF2BP2	Oncogene	MYC	E6/E7 promote CC by regulating MYC methylation sites via activating IGF2BP2	In vitro; in vivo	[133]
*Readers*					
IGF2BP2	Oncogene	circARHGAP12	CircARHGAP12 interacts with IGF2BP2 to combine with FOXM1 mRNA, thereby accelerating the stability of FOXM1 mRNA	In vitro; in vivo	[127]
YTHDF1/ IGF2BP3	Oncogene	PDK4	The m^6^A modified 5′UTR of PDK4 positively regulates its translation elongation and mRNA stability via binding with YTHDF1/eEF-2 complex and IGF2BP3, respectively	In vitro; in vivo	[116]
IGF2BP3	Oncogene	ACIN1	METTL3 interacts with IGF2BP3 to promote the mRNA stability of ACIN1	In vitro; in vivo	[100]
IGF2BP3	Oncogene	KCNMB2-AS1	KCNMB2-AS1 and IGF2BP3 form a positive regulatory circuit that enlarge the tumorigenic effect of KCNMB2-AS1 in CC	In vitro; in vivo	[125]
*Erasers*					
FTO	Oncogene	E2F1; MYC	FTO regulates CC cells proliferation and migration via controlling m^6^A modification of E2F1 and MYC transcripts	In vitro	[137]
FTO	Oncogene	β-catenin	FTO enhances the chemo-radiotherapy resistance through regulating expression of β-catenin by reducing m^6^A levels in its mRNA transcripts and in turn increases ERCC1 activity	In vitro; in vivo	[136]
FTO	Oncogene	HOXC13-AS	FTO-stabilized HOXC13-AS epigenetically up-regulates FZD6 and activates Wnt/β-catenin signaling	In vitro; in vivo	[135]
*Immunoregulators*					
METTL3	NA	NA	METTL3 expression is positively related to the density of CD33+ MDSCs in the TME	In-vitro	[150]

*NA* not available

### Role of m^6^A regulators in the progression of CC

#### m^6^A writers and CC

miRNA is a small non-coding RNA (18–24 nucleotides long), which negatively regulates the gene expression by binding to the 3′-UTR of its target mRNA at the post-transcriptional stage [94]. The correlations of miRNA activity with numerous physiological and pathophysiological processes, including carcinogenesis, have been reported [95, 96]. It was reported that the miRNAs might regulate over 60% of the human mRNAs and participate in almost all the biological processes in mammalian systems [97]. The functional characteristics of the miRNA target network and identification of miRNA dysregulation emphasized their importance in malignant tumors [98]. In CC, studies have shown that the m^6^A methylation modification might regulate the maturation of miRNA, thereby affecting the progression of tumors. Huang et al. reported that METTL3 could modulate the maturation of miR-193b by increasing the methylation level of pri-miR-193b m^6^A [99]. In addition, miR-193b might play a tumor suppressor role in the CC by inhibiting the cell cycle at the G1 phase as well as inhibiting the proliferation of cells [99]. Recently, Su et al. reported that METTL3 could stimulate the proliferation of CC by regulating mRNA stability of apoptosis chromatin condensation inducer 1 (ACIN1) mRNA [100]. Further investigations showed that the overexpression of IGF2BP3 could reverse the mRNA and protein levels of ACIN1 in the METTL3-downregulated CC cells, suggesting that the METTL3 could affect the mRNA stability of ACIN1 through IGF2BP3 [100].

In the previous section, it was elucidated that METTL3 could promote EMT in the OC by stimulating the mRNA translation of AXL [30]. Li et al. reported that METTL3 could negatively regulate the expression and membrane localization of β-catenin (encoded by CTNNB1) in the CC, thereby forming a complex with E-Cad to participate in the EMT and promoting the development of CC [101]. Further studies indicated that METTL3 could negatively regulate mRNA transcription, decay, and translation of CTNNB1 through different m^6^A regulators and mechanisms [101]. METTL3 indirectly inhibited the CTNNB1 transcription by upregulating the expression of its inhibitor E2F1 and recruiting YTHDF2 to the 5'-UTR m^6^A of CTNNB1 [101]. Additionally, METTL3 regulated both the classical and non-classical mRNA translation of CTNNB1 through YTHDF1 [101].

Piwi-interacting RNA (piRNA) was first discovered in 2006 as a small non-coding RNA, consisting of about 30 nucleotides, which could specifically bind to the PIWI family proteins (highly conserved RNA binding proteins) in the testicular germ cells [102, 103]. A recent study demonstrated that it was highly expressed both in the normal and cancer cells [104]. Their presence in the cancer cells might affect cancer growth by directly binding to the PIWI proteins [105]. Xie et al. reported the piRNA-14633-METTL14-CYP1B1 signaling cascade and showed the interaction of piRNA-14633 with the 3'-UTR of METTL14, which increased its mRNA stability, thereby enhancing the METTL14 methylase activity and promoting tumorigenesis by enhancing the CYP1B1 expression [106].

In OC, circRNA might act as a pro-oncogene and participate in the occurrence and development of tumors by promoting autophagy [57]. In CC, Chen et al. reported that the m^6^A regulator METTL3 could enhance the stability of circ0000069 transcripts, thereby showing the carcinogenesis effects of circRNA [107]. This resulted in the production of low levels of miR-4426 in the CC cells due to the enhanced circ000006 9[107]. Therefore, through m^6^A modification, miR-4426 was indirectly inhibited, which promoted CC development [107].

The methylation modifications of lncRNA and m^6^A play a key role in human cancer. The interaction mechanism between the lncRNA and m^6^A methylation modifications has been elucidated in other tumors, such as non-small cell lung cancer and CRC; however, little is known about their interaction in CC [108, 109]. Therefore, researchers have attempted to explore the internal mechanism of methylation modification of lncRNA and m^6^A, regulating the tumorigenesis of CC. Ji et al. reported a significantly upregulated expression of METTL3-induced FOXD2-AS1 in the CC cells and tissues, which could stabilize its mRNA [110]. FOXD2-AS1 could bind to the promoter region of p21 and recruit lysine-specific demethylase 1(LSD1) to silence its transcription, thereby accelerating the progression of CC [110]. In conclusion, they suggested that the METTL3/FOXD2-AS/LSD1/P21 axis could accelerate the CC progression in an m^6^A-dependent manner [110]. Yang et al. reported the role of m^6^A modification between miRNA and lncRNA and showed that the m^6^A modification significantly enriched the lncRNA ZFAS1 [111]. Further investigations showed the correlation of METTL3 with ZFAS1, interacting with the m^6^A level of ZFAS1 without affecting its expression level [111]. The knockdown of METTL3 abolished the miR-647-mediated suppression of ZFAS1, indicating that ZFAS1 could affect the miR-647 in an m^6^A-dependent manner [111]. These studies revealed that the lncRNA could promote the occurrence of CC in an m^6^A-dependent manner, and the lncRNA blocked the miRNA through the m^6^A modification-related proteins, thereby establishing a link between them and suggesting the potential mechanism of m^6^A between miRNA and lncRNA in CC.

Warburg effect, also known as aerobic glycolysis, is a typical metabolic marker of cancer metabolism [112–114]. Despite the abundance of intracellular oxygen, tumor cells persist to generate energy through aerobic glycolysis rather than oxidative phosphorylation through mitochondria [115]. This characteristic energy supplementation is known as the Warburg effect. Inhibiting the Warburg effect is considered an effective treatment for CC. The effects of m^6^A methylation modification on aerobic glycolysis in cancer cells have rarely been studied. Recent studies have investigated the molecular mechanism of m^6^A methylation modification in the energy metabolism in tumor cells. Li et al. reported the upregulated expression of pyruvate dehydrogenase kinase 4 (PDK4) induced by m^6^A, which was reversed by the METTL3 deficiency-induced inhibition of glycolysis and ATP production in the tumor cells [116]. A previous study reported that METTL3 was highly expressed in the metastatic tissues of CRC and inhibited the mRNA degradation of SRY-box transcription factor 2 (SOX2) by specifically interacting with m^6^A reader IGF2BP2 [117]. Similarly, Li et al. found that m^6^A modification in the 5′-UTR region rather than 3′-UTR of PDK4 mRNA could positively regulate its translation and stability by binding to the YTHDF1/eEF-2 complex and IGF2BP3 [116]. In addition, the TATA-binding protein (TBP) could enhance the expression of METTL3 in the CC cells [116]. The in-vivo and clinical analyses demonstrated that the m^6^A could regulate the glycolysis of cancer cells by regulation of PDK4 [116]. In another study, Wang et al. elucidated that METTL3 could promote the occurrence of CC in-vivo and in-vitro by promoting cellular glycolysis [118]. Meanwhile, the authors also showed that the m^6^A readers could stabilize mRNA, playing a carcinogenic role in the development of CC [118]. YTHDF1 could recognize HK2 m^6^A and enhance its stability, thereby regulating the HK2 mRNA [118]. METTL3 stabilized the HK2 mRNA by recruiting YTHDF1 and exerted an oncogenic effect via the YTHDF1/HK2 axis by accelerating glycolysis [118].

#### m^6^A reader and CC

In CC, studies on the m^6^A readers have mainly focused on the YTHDF1, YTHDF2, and IGFBPs. Previous studies showed that m^6^A readers could promote the progression of tumors by regulating mRNA translation [119]. Similarly, Wang et al. reported an elevated expression level of YTHDF1, which was closely related to the poor prognosis of CC patients [120]. RANBP2 was identified as a crucial target of YTHDF1 in the CC cells; YTHDF1 affected the RANBP2 translation in an m^6^A-dependent manner without affecting its transcription level [120]. Consequently, the overexpression of RANBP2 enhanced the progression of CC [120].

The lncRNAs have several functional roles, such as the organization of nuclear architecture, regulation of translation, mRNA stability, translation, and post-translational modifications [121, 122]. Currently, lncRNAs are reported to be deregulated in non-small cell lung cancer and CRC and play a tumor suppressor or oncogene function by inhibiting the miRNAs [123, 124]. Studies have also demonstrated the interaction of lncRNA with the m^6^A-related factors, affecting the occurrence and development of tumors. In CC, m^6^A readers promoted the growth and proliferation of the CC cells by affecting the fate of lncRNA [125, 126]. Zhang et al. reported that KCNMB2-AS1 was predominantly located in the cytoplasm and served as a ceRNA to inhibit the binding of miR-130b-5p and miR-4294 to the IGF2BP3 mRNA, resulting in its upregulation; therefore, it is a well-documented oncogene in CC [125]. Moreover, IGF2BP3 could bind to KCNMB2-AS1 through its three m^6^A modification sites acting as an m^6^A reader and stabilizing KCNMB2-AS1 [125]. The KCNMB2-AS1 and IGF2BP3 formed a positive regulatory circuit, which enhanced the tumorigenic effects of KCNMB2-AS1 in the CC [125]. Similarly, another study showed that the degradation of m^6^A-mediated GAS5 RNA relied on the YTHDF2 [126].

The circRAB11FIP1 might promote tumor autophagy in OC by regulating the m^6^A methylation of the autophagy-related proteins via FTO [57]. Similarly, Ji et al. showed that IGF2BP2 could interact with circARHGAP12, enhancing the mRNA stability of FOXM1 and forming a circARHGAP12/IGF2BP2/FOXM1 complex to promote the proliferation and migration of CC cells [127].

Human Papillomavirus (HPV) exists in several types, each of which, contains a circular double-stranded DNA genome. HPV primarily infects the basal keratinocytes of the squamous epithelium, which is poorly differentiated. The most common genotypes of HPV are HPV16/18, which are the high-risk types [128]. Generally, CC results from the persistent infection of high-risk HPV [129]. The HPV infection alters the metabolism of tumor cells, causing immune suppression and immune evasion, which lead to the occurrence of CC [130]. The alteration in metabolic phenotypes after HPV infection, contributing to the progression of malignant CC is a critical factor [131, 132]. Hu et al. elucidated the roles and mechanisms, underlying the biological effects of HPV E6/E7 and IGF2BP2 on the CC progression in-vitro and in-vivo [133]. They demonstrated that the E6/E7 proteins could stimulate aerobic glycolysis, proliferation, and metastasis in the CC cells by modulating the MYC mRNA via IGF2BP2 [133]. The E6/ E7 of HPV16 could enhance the expression of HK2 in glycolysis by increasing c-MYC [134]. These studies suggested complex correlations among the E6/E7, m^6^A methylation modification, promotion of aerobic glycolysis, and CC progression, the mechanisms of which require further studies.

#### m^6^A eraser and CC

In the CC, studies on the m^6^A eraser have focused on the FTO and ALKBH5 [126, 135–137]. The m^6^A demethylases play a critical role in the CC, including the interaction of FTO with the transcription factors E2F1 and MYC, which significantly reduces their translational efficiency [137]. The overexpression of E2F1 or MYC can compensate for the lack of FTO, which negatively impacts the proliferation and migration of cells, suggesting that these two genes might have mutual regulatory functions in CC cells [137].

Researchers elucidated that the lncRNA GAS5-AS1 could increase the expression and stability of GAS5 through YTHDF2 [126]. They reported that GAS5-AS1 could reduce the m^6^A level of GAS5 by interacting with m^6^A eraser ALKBH5, thereby increasing the stability of GAS5 [126]. Wang et al. suggested that FTO could regulate the fate of lncRNA to promote the development of CC [135]. They demonstrated that the reduction of the m^6^A level could improve the stability of HOXC13-AS in the CC cells [135]. Further investigations in the same study revealed that the HOXC13-AS promoted the epigenetically-mediated upregulation of FZD6 and activation of Wnt/β-catenin for promoting CC proliferation, invasion, and EMT [135].

Chemoradiotherapy is a major therapeutic option in CC treatment [138–140]. However, both the acquired and primary resistances to chemoradiotherapy cause treatment failure [140, 141]. Therefore, the mechanism of resistance to chemoradiotherapy in CC is needed to be investigated. Numerous studies showed that EMT was involved in drug resistance in cancer [142–145]. FTO could positively regulate the expression levels of β-catenin by reducing the m^6^A methylation level of its mRNA in EMT [136]. In the OC, the upregulation of the Wnt/β-catenin signaling pathway in EMT involved m^6^A modification [135, 146]. Zhou et al. also screened the markers of the Wnt/β-catenin pathway and demonstrated that the canonical Wnt/β-catenin pathway was not involved in the FTO-induced upregulation of β-catenin [136]. However, the excision repair cross-complementation group 1 (ERCC1) was determined as a downstream regulator of the FTO-induced up-regulation of β-catenin, confirming that FTO/β-catenin/ERCC1 axis might play an important role in developing resistance to the chemotherapeutic drugs in CC [136].

### m^6^A and immunoregulators in CC

The immunosuppressive cells in the TME, such as regulatory cells and MDSCs, affect each other as well as the development of tumors [147, 148]. Tumor-infiltrating MDSCs usually induce anti-tumor immune tolerance by inhibiting the proliferation and function of T cells, such as blocking the antigen presentation by the antigen-presenting cells [149]. Ni et al. reported that METTL3 could directly induce the differentiation of MDSCs and tumor-associated MDSCs *in-vitro*, suggesting that METTL3 might play an important role in the TME of CC [150].

Programmed death-1 (PD-1) is present in apoptotic T-cell hybridomas. It is predominantly present on the surface of activated T cells and B cells as a surface receptor for the activation of T cells. There are two ligands for PD-1, including PD-ligand 1 (PD-L1) and PD-ligand 2 (PD-L2) [151, 152]. TME enhances the expression levels of PD-1 molecules in the infiltrating T cells as well as those of PD-L1 and PD-L2 molecules in the tumor cells, thereby leading to constant activation of the PD-1 pathway within the TME. Zhang et al. investigated the effects of m^6^A-related lncRNA modifications on the immune response of CC patients [153]. The results showed a significant increase in the expression levels of several immune checkpoints in the high-risk subgroups associated with m^6^A, including PD-1 and PD-L1, which suggested the potential responses to PD-1 [153].

### Prognostic effects of m^6^A in CC

The diagnosis and treatment of cancer have been greatly improved over the past decades. However, the 5-year survival rate of the patients remains low. Therefore, accurate prognostic indicators are needed to establish an individualized treatment strategy for CC patients (Table 4). Wang et al. reported that a decrease in the m^6^A methylation level was closely related to the cancer progression and low survival rate, suggesting its potential as a target for the prognosis and treatment of CC [154]. Ma et al. reported that four genes had the prognostic potential for CC, including HNRNPC, KIAA1429, and ZC3H13, and a protective gene YTHDF1 [155]. Pan et al. established a characteristic model, consisting of three genes (ZC3H13, YTHDC1, and YTHDF1), and showed good performance in predicting the survival rates of cervical squamous cell carcinoma (CESC) patients [156]. Moreover, the results were validated using bioinformatics analysis in clinical cohort of CESC [156]. The protein and mRNA expression levels of ZC3H13, YTHDC1, and YTHDF1 were detected in further experiments [156]. The experimental results were consistent with the in-silico results, confirming their prognostic potential in CESC [156].

## m^6^A and EC

**Fig. 3:**
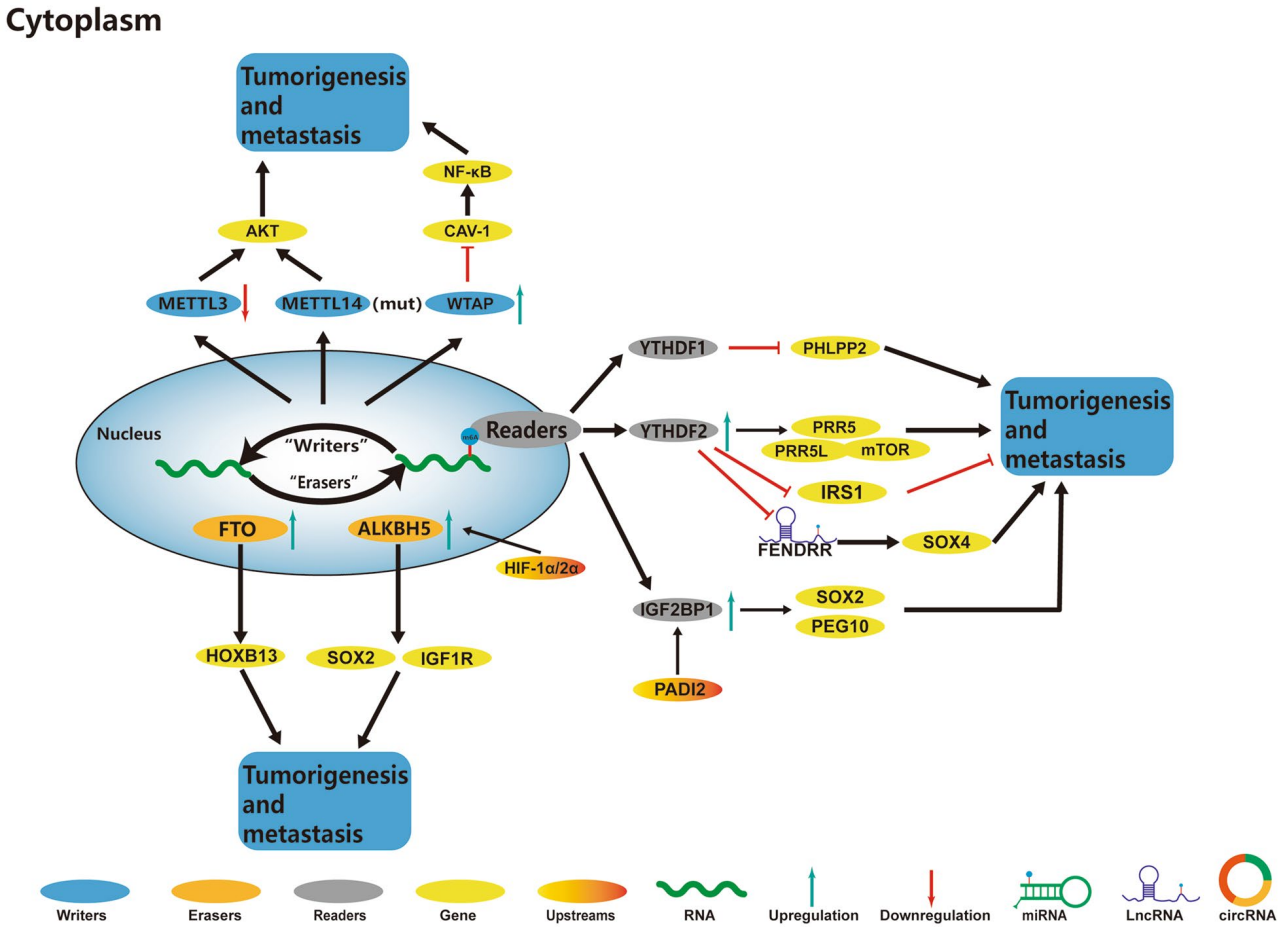
In EC, m^6^A regulatory proteins contribute to tumorigenesis and metastasis by interacting with various RNAs. METTL14 mutation or reduced expression of METTL3 increases the proliferation and tumorigenicity of EC by activating the AKT pathway. WTAP downregulates CAV‐1 expression to activate the NF‐κB signaling pathway in EC, promoting EC progression. HIF-1α and HIF-2α activate the expression of ALKBH5 under hypoxic conditions, facilitating the SOX2 expression by demethylating the SOX2 mRNA, leading to the tumorigenesis of EC. ALKBH5 demethylates the target transcript IGF1R and enhances its mRNA stability to promote tumorigenesis and metastasis of EC. FTO promotes HOXB13 protein expression, activates the WNT signaling pathway, and promotes EC invasion and metastasis. PADI2 activates the IGF2BP1 expression and helps in maintaining the mRNA stability and expression of SOX2, thereby supporting the malignancy state of EC. IGF2BP1 recruits PABPC1 to promote PEG10 protein expression, contributing to the tumorigenesis of EC. YTHDF2 inhibits the expression of IRS1 and inhibits IRS1/AKT signaling pathway, consequently inhibiting the tumorigenicity of EC. YTHDF2-mediated LncRNA FENDRR degradation promotes cellular proliferation by elevating the SOX4 expression in EC

Numerous studies have explored the participation of m^6^A regulators in many functions in the EC, such as cell cycle regulation and self-renewal of CSCs. The role and mechanism of m^6^A regulators in EC are summarized in Fig. 3 and Table 3.

**Table 3:** The role and mechanism of m^6^A regulators in EC

m^6^A regulators	Roles	Genes/RNAs	Mechanisms	Model	Refs.
*Writers*					
METTL3/METTL14	Tumor suppressor	PHLPP2; mTORC2	METTL14 mutation or reduced METTL3 reduces the level of m^6^A methylation, leading to decrease expression of the negative AKT regulator PHLPP2 and increase expression of the positive AKT regulator mTORC2	In vitro; in vivo	[168]
WTAP	Oncogene	CAV-1	WTAP methylates 3′‐UTR of CAV‐1 and downregulates CAV‐1 expression to activate NF‐κB signaling pathway	In vitro; in vivo	[159]
*Readers*					
YTHDF2	Oncogene	FENDRR	YTHDF2-mediated LncRNA FENDRR degradation promotes cell proliferation by elevating SOX4 expression	In vitro; in vivo	[170]
YTHDF2	Tumor suppressor	IRS1	YTHDF2 binds the methylation sites of target transcripts IRS1 and promotes IRS1 mRNA degradation, consequently inhibiting the expression of IRS1 and inhibiting IRS1/AKT signaling pathway	In vitro	[169]
IGF2BP1	Oncogene	PEG10	IGF2BP1 can recognize m^6^A sites in the 3′UTR of PEG10 mRNA and recruit PABPC1 to enhance PEG10 mRNA stability, which consequently promotes PEG10 protein expression	In vitro; in vivo	[176]
IGF2BP1	Oncogene	SOX2	Dysregulation of IGF2BP1 by PADI2/MEK1/ERK signaling results in abnormal accumulation of oncogenic SOX2 expression	In vitro; in vivo	[183]
*Erasers*					
FTO	Oncogene	HOXB13	FTO demethylates m^6^A modifications in HOXB13 mRNA and promotes EC metastasis by activating the WNT signaling pathway	In vitro; in vivo	[146]
ALKBH5	Oncogene	IGF1R	ALKBH5 demethylates target transcripts IGF1R and enhances IGF1R mRNA stability, consequently promoting IGF1R translation and activating IGF1R signaling pathway	In vitro	[189]
ALKBH5	Oncogene	SOX2	ALKBH5 in promoting SOX2 transcription via mRNA demethylation, thereby maintaining the stem-like state and tumorigenicity potential of ECSCs	In vitro; in vivo	[197]
*Immunoregulators*					
ZCH3H13, METTL14, and YTHDC1	NA	NA	the expression, mutation, and SCNAs of these genes are associated with the immune cell infiltration	NA	[198]

*NA* not available

### Role of m^6^A in the progression of EC

#### m^6^A writer and EC

The signal transduction network is a communication line among cells, and is used for perceiving signals, including those from the extracellular environment, and transmitting them to the downstream targets for the proper functioning and maintenance of cells. The abnormal changes in the signal transduction pathways are the important biological characteristics of the tumor cells. Current studies have shown that the alternation in the signal transduction pathways affects the cellular metabolism and immune response in the tumor cells [157, 158]. In EC, the mechanism of m^6^A methylation effects on tumor growth through signaling pathways has been elucidated. Liu et al. reported higher expression levels of WTAP in the EC tissues as compared to the adjacent normal tissues [159]. Their further investigations confirmed that the expression of CAV-1 was regulated by WTAP in an m^6^A-dependent manner [159]. They also showed that, after being regulated by WTAP, the CAV-1 could activate the downstream NF-κB pathway [159].

PI3K/AKT pathway also plays an important role in various biological processes. The dysregulation of the AKT signaling pathway has shown crucial roles in the proliferation and apoptosis of numerous tumor cells [160–162]. Some studies showed that the stem cells and cancer cells proliferated with the reduction in the m^6^A methylation [36, 163, 164]. However, other studies reported that some cancers were related to the high expression of METTL3 and increased m^6^A methylation, which might involve different mechanisms and require more in-depth and detailed studies [35, 165, 166].

#### m^6^A reader and EC

Researchers have shown that the dynamic m^6^A-modification in mRNAs, particularly in the key transcripts, might alter the physiology of cells [167]. For instance, the decreased m^6^A mRNA methylation could stimulate cellular proliferation by modulating the expression of the critical enzymes, which were involved in the AKT signaling pathway [168]. Hong et al. showed that the overexpressed YTHDF2 could bind to the m^6^A-modified insulin receptor substrate 1 (IRS1), which reduced its translation, thereby blocking the IRS1/AKT pathway [169]. In the same study, the results indicated that several vital proteins could regulate the cellular activities of EC cells through the AKT pathway; these proteins were also responsible for regulating the dynamic equilibrium of EC cells [169].

On the other hand, studies showed that YTHDF2 could also regulate the proliferation of EC cells by affecting the metabolism of lncRNA. According to Shen et al., the expression levels of lncRNA FENDRR in the EC tissues were reduced; however, its m^6^A methylation levels showed a negative effect trend [170]. The subsequent *in-vitro* experiments in the same study demonstrated that YTHDF2 could recognize the abundance of m^6^A-modified lncRNA FENDRR in the EC cells and degrade it [170]. After expressing the YTHDF2 gene, the expression levels of lncRNA FENDRR were restored, thereby inhibiting proliferation and stimulating the apoptosis of EC cells [170]. Furthermore, they reported that overexpressing the lncRNA FENDRR could reduce SOX4 translation and result in inhibiting EC cell proliferation and promoting cellular apoptosis [170]. These results were consistent with those of the previous study by Liu et al., which reported an adverse effect of the lncRNA FENDRR on the SOX4 expression in CRC [171].

Among the molecular mechanisms of cellular proliferation, cell cycle acceleration is of great importance, which is regulated by the CDK-cyclin complexes and cyclin-dependent kinase inhibitors [172]. A recent study reported that paternally expressed gene 10 (PEG10), a critical factor, which directly modulates the key proteins, was involved in cell cycle proteins [173]. Numerous studies have demonstrated the contributions of PEG10 to cellular proliferation; however, its mechanism has rarely been studied [174, 175]. The knockdown of the PEG10 increased the p21 and p27 expression levels [173]. Zhang et al. reported that IGF2BP1 could recognize the m^6^A site in the 3′-UTR of the PEG10 mRNA in EC and recruit the polyadenylate binding protein 1 (PABPC1) to stabilize PEG10 mRNA, thereby increasing its protein expression levels and accelerating the cell cycle [176].

The peptide arginine deaminases (PADIs) family contains five members, including PADI1-4 and PADI6. Except for the PADI6 which has no enzymatic activity and is expressed only in the ovary [177], other PADIs can deaminate positively charged arginine residues in substrate proteins into the neutral non-coding residues called citrulline [178, 179]. The expression of PADIs was higher in various malignant tumor tissues as compared to healthy tissues [180, 181]. Numerous studies showed that the PADIs-catalyzed protein citrullination could alter signal transduction, cell differentiation, and EMT in a variety of human cancer cells [180, 182]. Xue et al., for the first time, reported that m^6^A reader IGF2BP1 could be used as a downstream factor of PADI2 and could regulate it to promote tumor progression in EC [183]. They further showed that PADI2 could interact with MEK1 kinase in the MAPK pathway and catalyze the citrullination, which was beneficial for the phosphorylation of ERK1/2 by MEK1, thereby activating the expression of IGF2BP1. In addition, IGF2BP1 could also bind to the m^6^A site in the 3′-UTR of SOX mRNA to prevent its degradation [183]. This study revealed that the PADI2/MEK1/ERK/IGF2BP1 pathway could promote the characteristics of carcinogenic tumor cells in EC, and the combination of specific PADI2 and MEK1 inhibitors might provide a novel therapeutic target site for the treatment of the MEK inhibitor-resistant EC patients [183].

#### m^6^A eraser and EC

The insulin-like growth factor (IGF) is involved in many functions in most organs [184–186]. IGF1 and IGF2 can affect EC, as observed in both clinical and experimental data [187]. Both the IGF1 and IGF2 ligands can activate insulin-like growth factor 1 receptor (IGF1R), a tyrosine kinase receptor present on the cell surface, which is coupled with several intracellular secondary messenger pathways, including Ras/Raf/MAPK and PI3K/AKT signaling pathways, especially in regulating the normal uterine physiology [188]. Pu et al. reported that ALKBH5 enhanced the stability and translation of IGF1R mRNA by the demethylation of m^6^A and increased the protein levels of COL1A1 and MMP9, thereby promoting the tumorigenesis of EC cells [189]. However, the possibility of involving other signaling pathways cannot be excluded. Other signaling pathways may alter either directly or indirectly due to the changes in ALKBH5 and require further investigation.

The role of m^6^A methylation, participating in the Wnt signaling pathway by regulating the related RNAs or proteins, has been elucidated in the CC [56, 135, 136]. FTO could remove the m^6^A modification of HOXB13 mRNA, abolish the degradation of HOXB13 mRNA mediated by YTHDF2, promote the expression of HOXB13 protein, activate the Wnt signaling pathway, and promote the invasion and metastasis of EC [146].

Hypoxia is an important niche feature of the CSCs, positively affecting the growth of stem cells and tumor progression [190, 191]. Hypoxia-inducible factors (HIFs), including HIF-1α and HIF-2α, are the main media of hypoxia and indispensable for the activation and self-renewal of CSCs; they are strongly associated with tumors [192]. The ability of CSCs to tolerate hypoxia can be attributed to the rearrangement of genes involved in cellular multipotency and differentiation [193]. All these studies further deepen the understanding of the correlations among hypoxia, HIFs, and SOX2 [194–196]. Chen et al. demonstrated that the hypoxia and high levels of ALKBH5 could restore the stemness of differentiated endometrial CSCs (ECSCs) and increase the ECSC-like phenotype [197]. In addition, a recent study showed that the changes in mRNA stability were negatively correlated with the expression of these multipotency factors [65]. The m^6^A reader IGF2BP1 could stabilize the degradation of SOX2 mRNA in EC, thereby promoting tumor progression [183]. Similarly, Chen et al. verified that ALKBH5 could stimulate SOX2 mRNA expression by reducing its m^6^A methylation level, thereby increasing the stemness and carcinogenicity of ECSCs [197]. These studies revealed that the decrease in the m^6^A mRNA methylation in the key mRNAs might be a potential mechanism of most EC. These studies also confirmed that m^6^A methylation was a regulatory factor for cell growth.

### m^6^A and immunoregulation in EC

In EC, the correlation between m^6^A methylation modification and TME has rarely been reported. Recently, Ma et al. analyzed the EC patients’ data from TCGA and identified the genetic changes in the m^6^A regulatory genes. The results showed a significant correlation between the negative changes in the m^6^A levels and adverse prognostic outcomes [198]. The study identified ZC3H13, METTL14, and YTHDC1 as independent prognostic factors for EC patients [198]. Noteworthy, the expression, mutation, and somatic copy number alterations (SCNAs) of these genes were associated with immune cell infiltration [198].

### Prognostic effect of m^6^A in EC

The incidence of EC has increased over the past few decades, making it one of the most prevalent gynecologic cancers [199]. In 2020, 417,367 new cases of EC were diagnosed, causing 97,370 deaths [1]. The global incidence rate of EC is continuously increasing, while those of several other types of cancer have decreased over the past two decades [200–204]. Despite the better prognosis of EC than that of CC and OC, screening for the high-risk parts of EC patients is imperative due to more likeliness of developing advanced cancer and early death. With the advancements in the studies on m^6^A methylation modification in EC, numerous research groups have reported the prognostic potential of m^6^A regulatory factors and m^6^A-related genes in EC (Table 4). In a recent study, Zhai et al. analyzed the TCGA dataset of EC patients and established a risk model based on the m^6^A regulators, particularly FTO, RBM15, and YTHDF1, and revealed their crucial roles in the development and prognosis of EC [205]. Further analysis of the datasets showed that FTO and RBM15 could affect the survival of EC patients by regulating the m^6^A-associated genes involved in the development of connective tissue, catabolism, RNA stability, oxidative demethylation, temperature balance, and energetic metabolism [205]. Zhang et al. established a reliable protective model based on seven significant CpG sites located in the m^6^A regulators. The model could effectively predict the prognosis of EC, indicating that the CpG sites might be advantageous in predicting the EC [206]. Three m^6^A-associated lncRNAs revealed by Shi et al. that, in contrast to the TRAF3IP2-AS1, AL133243.2, the patients with the high SCARNA9 expression tended to have a worse prognosis [207]. These lncRNAs were verified to participate in the development of endometriosis by modulating m^6^A-related enzymes, suggesting that these RNAs might be associated with the diagnosis and treatment of EC [207].

**Table 4:** The prognostic potential of m^6^A regulatory genes and m^6^A associated genes

Prognostic genes/model	Univariate Cox Analyses		Multivariate Cox Analyses		Sample size	Refs.
	HR (95 CI%)	*P*	HR (95 CI%)	*P*		
*OC*						
WTAP	NA	*P* = 0.021	1.191 (1.023–1.385)	*P* = 0.024	231	[84]
YTHDC2 and KIAA1429	3.143 (1.516–6.517)	*P* = 0.002	2.330 (1.116–4.865)	*P* = 0.024	379	[88]
IGF2BP1, VIRMA and ZC3H13	1.25 (1.10–1.43)	*P* < 0.001	1.24 (1.08–1.41)	*P* = 0.002	379	[86]
VIRMA, IGF2BP1, and HNRNPA2B1	1.62 (1.20–2.20)	*P* = 0.002	1.60 (1.18–2.17)	*P* = 0.003	374	[87]
WTAP, LGR6, ZC2HC1A, SLC4A8, AP2A1, NRAS, CUX1, HDAC1, CD79A, ACE2, FLG2, and LRFN1	1.664 (1.481–1.868)	*P* = 8.5E-18	1.699 (1.508–1.913)	*P* = 2.29E-18	373	[89]
*CC*						
HNRNPC, KIAA1429, ZC3H13	2.268 (1.161–4.431)	*P* = 0.017	2.583 (1.289–5.174)	*P* = 0.007	306	[155]
ZC3H13, YTHDC1, and YTHDF1	NA	NA	4.592 (2.788–7.562)	*P* < 0.001	304	[156]
*EC*						
FTO, RBM15, and YTHDF1	1.129 (1.065–1.197)	*P* < 0.001	1.090 (1.023–1.161)	*P* = 0.008	406	[205]
7 CpG sites (cg13823621, cg08881614, cg07867023, cg22247039, cg00624976 cg06778680 and, cg13204529)	NA	NA	4.3 (2.4–7.6)	*P* < 0.001	312	[206]

*NA* not available

## Conclusions and perspectives

In this review, the studies on the m^6^A methylation modification in OC, CC, and EC were summarized from the aspects of tumor development, immune microenvironment, and prognosis. From the existing studies, it was concluded that the abnormal expression of the m^6^A regulator in gynecological tumors might lead to an increase or decrease in the m^6^A modification level of RNA. The m^6^A modifications of RNA might affect the fate of various RNAs and lead to the proliferation, invasion, and metastasis of tumors as well as also alter the tumor immune microenvironment of the patients, thereby participating in the occurrence and development of tumors.

The advancements in medical technology have greatly improved the survival rate of gynecologic cancer patients than before. However, due to the lack of specific biomarkers, early diagnosis is still challenging. At the same time, the resistance to the anti-tumor drugs in some patients also urges researchers to deepen the understanding of tumorigenesis and identify new immune targets for the development of anti-tumor drugs. This review summarized the results of numerous studies, which showed that the m^6^A regulator and related genes could be used as potential biomarkers or prognostic indicators for the early diagnosis of gynecologic cancers. Studying the mechanism of the m^6^A regulator in tumor development also supported this view. The role of m^6^A modification in the drug resistance mechanism has also been reported, which showed that the inhibitors of m^6^A regulators might have the potential of being used as anti-tumor drugs in drug-resistant patients.

At present, numerous studies have reported the mechanism of m^6^A-promoting effects on the development of gynecologic cancer; however, the knowledge is still insufficient for a deeper understanding of tumorigenesis. The mechanisms, explaining the upregulation of the m^6^A regulator in gynecologic cancer and their relationship with oncogenes, are still unclear. Therefore, further studies are needed in the future to explain these mechanisms in order to develop effective therapeutic strategies.

## Data Availability

Not applicable.

## Abbreviations

3′-UTR: 3′-Untrasnlated region
5′-UTR: 5′-Untranslated region
ACIN1: Apoptosis chromatin condensation inducer 1
ALKBH5: AlkB homolog 5
AML: Acute myeloid leukemia
AXL: AXL receptor tyrosine kinase
CC: Cervical cancer
CDC42EP3: Cell division cycle 42 effector protein 3
ceRNA: Competing endogenous RNA
CESC: Cervical squamous cell carcinoma
circRNAs: Circular RNAs
CNV: Copy number variation
CRC: Colorectal cancer
CSC: Cancer stem cell
EC: Endometrial cancer
ECSCs: Endometrial cancer stem cells
EMT: Epithelial–mesenchymal transition
ERCC1: Excision repair cross-complementation group 1
FAM76A: Family with sequence similarity 76 member A
FBW7: F-box and WD repeat domain-containing 7
FTO: Fat mass and obesity-associated protein
FZD10: Frizzled class receptor 10
HBS1L: HBS1 like translational GTPase
HCC: Hepatocellular carcinoma
HGSOC: High-grade serous ovarian cancer
HIFs: Hypoxia-inducible factors
HOXA10: Homeobox A10
HPV: Human papillomavirus
IGF: Insulin like growth factor
IGF1R: Insulin-like growth factor 1 receptor
IGF2BPs: Insulin-like growth factor 2 mRNA-binding proteins
IRS1: Insulin receptor substrate 1
JAK2: Janus kinase 2
lncRNAs: Long noncoding RNAs
LSD1: Lysine-specific demethylase 1
m^6^A: N^6^-methyladenosine
MDSCs: Myeloid-derived suppressor cells
METTL14: Methyltransferase-like 14
METTL3: Methyltransferase-like 3
miRNAs: MicroRNAs
mRNA: Messenger RNAs
mTOR: Mammalian target of the rapamycin
NANOG: Nanog homeobox
ncRNA: Non-coding RNAs
OC: Ovarian cancer
PABPC1: Polyadenylate binding protein 1
PADIs: Peptide arginine deaminases
PARPi: PARP Inhibitor
PD-1: Programmed death-1
PDK4: Pyruvate dehydrogenase kinase 4
PD-L1: Programmed death ligand 1
PD-L2: Programmed death ligand 2
PEG10: Paternally expressed gene 10
PI3K: Phosphoinositide 3-kinase
piRNA: Piwi-interacting RNA
PTEN: Phosphatase and tensin homolog
RBM15: RNA binding pattern protein 15
rRNA: Ribosomal RNAs
SCNAs: Somatic copy number changes
SOX2: SRY-box transcription factor 2
SOX4: SRY-related HMG box transcription factor 4
STAT3: Signal transducer and activator of transcription 3
TCGA: The cancer genome atlas
TICs: Tumor-infiltrating immune cells
TLR4: Toll-like receptor 4
TME: Tumor microenvironment
TRIM29: Tripartite motif containing 29
TROAP: Trophinin associated protein
VIRMA/KIAA1429: Vir-like m6A methyltransferase-associated protein
WTAP: Wilm’s tumor-associated protein
YTHDC1: YTH domain containing 1
YTHDF1: YTH N6-methyladenosine RNA binding protein 1
YTHDF2: YTH N6-methyladenosine RNA binding protein 2
ZC3H13: Zinc finger CCCH domain-containing protein 13
